# Different Effects of Thermophilic Microbiological Inoculation With and Without Biochar on Physicochemical Characteristics and Bacterial Communities in Pig Manure Composting

**DOI:** 10.3389/fmicb.2021.746718

**Published:** 2021-11-16

**Authors:** Likun Sun, Min Long, Jianshu Li, Renfei Wu, Lin Ma, Defu Tang, Yongli Lu, Ziyu Wang

**Affiliations:** ^1^College of Animal Science, Gansu Agricultural University, Lanzhou, China; ^2^Gansu Provincial Engineering Research Center for Animal Waste Utilization, Gansu Agricultural University, Lanzhou, China; ^3^College of Veterinary Medicine, Gansu Agricultural University, Lanzhou, China; ^4^Key Laboratory of Agricultural Water Resources, Hebei Key Laboratory of Soil Ecology, Center for Agricultural Resources Research, Institute of Genetic and Developmental Biology, Chinese Academy of Sciences, Shijiazhuang, China; ^5^College of Resources and Environmental Sciences, Gansu Agricultural University, Lanzhou, China

**Keywords:** microbiological inoculation, biochar, pig manure composting, bacterial communities, physicochemical characteristics

## Abstract

This study evaluated the effects of thermophilic microbiological inoculation alone (TA) and integrated with biochar (TB) on the physicochemical characteristics and bacterial communities in pig manure (PM) composting with wheat straw. Both TA and TB accelerated the rate of temperature increase during the PM composting. TA significantly reduced total nitrogen loss by 18.03% as opposed to TB which significantly accelerated total organic carbon degradation by 12.21% compared with the control. *Firmicutes*, *Bacteroidetes*, *Actinobacteria*, and *Proteobacteria* were the major phyla in composting. Variation of the relative abundance of genera depended on the composting period and treatment. The genera *Lactobacillus* (26.88–46.71%) and *Clostridium_sensu_stricto* (9.03–31.69%) occupied a superior position in the temperature rise stage, and *Bacillus* (30.90–36.19%) was outstanding in the cooling stage. Temperature, total nitrogen (TN), and ammonium nitrogen significantly influenced the bacterial phyla composition. TN, water content, and nitrite nitrogen were the main drivers of the bacterial community genera. Furthermore, our results demonstrated that microbiological consortia were resistant to high temperatures and could fix nitrogen for enriched *Pseudomonas*; however, when interacted with biochar, total organic carbon (TOC) degradation was accelerated for higher bacterial richness and diversity as well as overrepresented *Corynebacterium*.

## Introduction

A rapid increase in the demand for pork products has resulted in the production of large amounts of pig manure (PM) in China. The figure released by the National Bureau of Statistics of China [NBSC] (2016) indicated that PM production approached nearly 490 million tons in 2015. PM contains harmful substances, such as heavy metals, unfavorable odors, parasites, and pathogens, which pose potential risks to the environment and public health, especially with improper treatment and application (Ravindran et al., 2019; Awasthi et al., 2020a). However, PM contains high amounts of organic matter and nutrients (nitrogen, phosphorus, and potassium), making it a valuable resource. Thus, it should be handled and managed properly before being applied to farmland as an organic fertilizer.

Aerobic composting is an important resource utilization process for organic waste. It is a complex biodegradation of a mixture of solid substrates conducted by microbial community composed of various populations under aerobic conditions (Maheshwari, 2014). Raw organic materials are mineralized and stabilized into biologically stable, humic-like substances (Maheshwari, 2014). Composting can also reduce pathogens, such as *Escherichia coli* and *Salmonella* spp., odor, and heavy metals (Agyarko-Mintah et al., 2017). However, traditional composting has been proved to be a slow process of decomposition.

Additives are a promising strategy for accelerating organic matter decomposition and improving compost potency. Biochar has been extensively studied because its porosity allows it to promote degradation, absorb gaseous NH_3_ (Mao et al., 2018; Duan et al., 2019a; Zhou et al., 2019), and reduce nutrient loss (Vandecasteele et al., 2016; Awasthi et al., 2017a,b; Godlewska et al., 2017). Biochars can also provide a habitat for beneficial microorganisms because of their large surface areas, which stimulate microbial activity (Duan et al., 2019a; Mao et al., 2019; Zhou et al., 2019).

Compost results from microbiological processes and organic matter decomposition strongly rely on the activity of microorganisms (Mao et al., 2018); therefore, maintaining a beneficial bacterial composition is important for composting. There have been many attempts to improve the degree of maturation and nutrient sequestration by inoculation with degrading microbiological strains, such as *Trametes versicolor*, *Thermoactinomyces* sp., *Trichoderma harzianum*, *Rhizopus oryzae*, *Bacillus* sp., and *Bacillus stearothermophilus* (Hachicha et al., 2012; Zhou et al., 2015; Kuroda et al., 2017; Wang et al., 2019) or lignocellulose-degrading and cellulose-degrading microflora (Wan et al., 2020; Yu et al., 2020). Recently, (Duan et al., 2019a,b) and Awasthi et al. (2020b) explored the dynamics of bacterial communities after biochar-integrated microbial inoculation on the degree of access degradation and greenhouse gas emissions during manure composting. These studies emphasized the influence of the interaction of bacterial cultures with different types of biochar (wheat straw, wheat straw biochar, and wood biochar), but the composition and function of the bacterial consortia were not clear. It is well known that microbiological communities that appear in compost result from intricate dynamic interactions between microbes and the composting environment in different phases. However, relationships between microbiological inoculation with and without biochar and the physicochemical environment during PM composting are still unknown.

In this study, microbiological inoculates mixed with thermophilic cellulose-degrading microorganisms and deodorizing microorganisms were used to explore the effects of PM compost inoculation. Furthermore, thermophilic microbiological inoculation was also integrated with biochar and the following was attempted: (1) to determine the influence of physicochemical characteristics; (2) to study changes in bacterial abundance, diversity, and community structures during composting; and (3) to evaluate interactions between bacterial composition and physicochemical properties to further explore the main role of microbial inoculum on PM composting.

## Materials and Methods

### Microbiological Inoculum

The microbiological inoculum was obtained from a laboratory, enriched from fresh pig dung and PM. The inoculum contained two types of microorganisms (1:1), thermophilic cellulose-degrading microorganisms (Cel3) and deodorizing microorganisms (BHC2). The Cel3 population was enriched by incubating 12.5-g samples in 250 mL of enrichment culture medium (CMC-Na 10 g/L, (NH_4_)_2_SO_4_ 4 g/L, KH_2_PO_4_ 2.0 g/L, MgSO_4_⋅7H_2_O 0.5 g/L, CaCl_2_ 0.3 mg/L, FeSO_4_⋅7H_2_O 1.6 mg/L, ZnSO_4_⋅7H_2_O 1.4 mg/L, pH 7.2). The medium was cultured for 3–5 days at 50°C at 120 rpm/min and transferred to 500 mL of an enrichment culture medium for 5–7 days. The microbial population was cultured for 3 months until microbial inoculum stability. A filter paper degradation experiment showed that Cel3 can degrade 72.82% of the filter paper in 7 days. BHC2 were enriched by incubating 12.5 g in 250 mL of an ammonia-selective medium (sucrose 5 g/L, ammonia water 10 mL/L, KH_2_PO_4_ 2 g/L, MgSO_4_⋅7H_2_O 0.5 g/L, FeSO_4_⋅7H_2_O 0.1 g/L, 1% ZnSO_4_ 5 mL/L, NaCl 2 g/L, nutrient broth 0.5 g/L) as Chen et al. (2016) described. The medium was cultured for 5–7 days at 35°C, at 150 rpm/min, then transferred to 500 mL of enrichment culture medium for 7 days. Bacterial enrichments were cultivated for 3 months, and the most capable of ammonia removal from the microbial community was selected. Compositions of the stable microbial communities Cel3 and BHC2 were analyzed by high-throughput sequencing using the bacterial 16s rRNA gene and fungal internal transcribed spacer (ITS) region. The results showed Cel3 included *Pseudomonas*, *Mortierella*, and *Cladosporium*, whereas BHC2 was comprised of *Bacillus* and *Brevibacillus.* Then, microorganisms were incubated for 3 days at a concentration of 10^7^ cells/mL to serve as a microbiological inoculum.

### Composting Process and Sampling

Fresh pig dung was obtained from a commercial pig plant near Lanzhou (Gansu). Wheat straw was obtained from the Ganzhou district (Zhangye, Gansu) and cut into 2–3 cm pieces. Coconut shell biochars—used as the additive in this experiment—were obtained from Huaibei Jieli Biotechnology Co., Ltd. (Jiangsu Province, China). Biochars were ground into a uniform size and passed through a 5-mm sieve before use. The details of the raw material properties are listed in Supplementary Table 1. The following three additives were uniformly mixed with the PM containing wheat straw: no additives in the control (CK); microbiological inoculum (microbiological inoculum volume/pile quality was 1%) in treatment A (TA), and 1% microbiological inoculum along with 10% coconut shell biochar in treatment B (TB). Fresh pig dung was mixed with wheat straw at a dry weight mass ratio of 4.5:1 and yielded the required water content of approximately 65% and a C/N ratio of approximately 22:1. The laboratory-scale experiment was conducted in a glass bottle (20 L) with a forced oxygen entry using an oxygen bottle. A schematic of the composting reactor is shown in Supplementary Figure 1. The outer wall of the glass bottle was insulated with wheat straw. During the 35 days of the composting process, the bottles were ventilated for 30 min twice daily at 120 L/h and were mixed continuously every 3 h in the daytime.

Compost samples were collected from each reactor during the initial, mesophilic, thermophilic, cooling, and maturity phases on days 2, 8, 16, 26, and 35 of the composting process. Approximately 0.5 kg of mixed samples from the surface, middle, and bottom layers were taken using five-point sampling and conducted with three replicates. Fresh samples were gently mixed and split into two parts. The first part was air-dried in the shade, passed through an 80-mesh sieve, and then used for physicochemical property analysis. The second part was stored at –80°C and used for 16S rDNA high-throughput sequencing.

### Measurement of the Physicochemical Characteristics of the Samples

Compost temperature changes were recorded daily. Temperature levels at the surface, middle, and bottom of the layers were measured three times using digital thermometers. The average temperature of each sample was used for the analysis. Water content was determined by the oven drying method. pH values were analyzed using a pH meter (PT-10, Sartorius, Göttingen, Germany). Electricity conductivity (EC) was determined using an EC meter (Kedida-CT-3030, China), and TN values were measured with an automatic element analyzer (Elementar Vario-EL, Germany). Total organic carbon (TOC) was measured using the potassium dichromate oxidation method with external heat (Sun et al., 2019). Total phosphorus (TP) concentrations were determined using colorimetry (UV-5100, Shanghai Yuanxi, China) after samples were treated with H_2_SO_4_ and H_2_O_2_. Total potassium (TK) content was determined using flame atomic absorption spectrophotometry (ZEEnit 700, Analytik Jena, Germany). Ammonium nitrogen (NH_4_^+^-N) was extracted using 2.0 mol/L of potassium chloride, and its concentration was estimated using colorimetry with indophenol blue. Nitrate nitrogen (NO_3_^–^-N) was extracted using 50% hydrochloric acid, and its concentration was determined by colorimetry (UV-5100, Shanghai Yuanxi, China). Three replicates were conducted for each physicochemical characteristic. The seed germination index (GI) is an index used to determine compost maturity and its toxicity to plants. Twenty *Brassica napus* seeds were placed on filter paper soaked in 5 mL of compost extract, obtained with a 1-g sample per 10 mL of distilled water, soaked in distilled water as a control. Each sample was replicated three times. After incubation at 25°C for 24 h, seed germination and root length were determined using the method described by Khan et al. (2014). GI was calculated according to Equation (1):


(1) $$GI\left(\%\right)=\frac{seedgerminationintreatment\left(\%\right)\times rootlengthintreatment}{seedgerminationincontrol\left(\%\right)\times rootlengthincontrol}\times 100$$

### DNA Extraction, Amplification, and HiSeq Sequencing

Total DNA was extracted from 0.25-g mixed samples using the Soil DNA Isolation Kit (TIANGEN DP336, China). High-throughput sequencing was used to study the variable V3–V4 region of 16S rRNA to analyze the changes in bacterial communities. The amplicons were sequenced using the Illumina HiSeq 2500 system (Illumina, United States) by Biomarker Co., Ltd. (Beijing, China).

### Bioinformatics Analysis

In total, 3,680,900 raw reads were merged by overlapping paired-end reads using Flash V1.2.7 and then were quality-trimmed using Trimmomatic v0.33 (Bolger et al., 2014). Chimeric sequences were removed using the UCHIME v4.2 (Edgar, 2018) program and effective tags were obtained. Operational taxonomic units (OTUs) were clustered at a 97% identity threshold using UPARSE (Edgar, 2013). Taxonomic annotations of the OTUs were determined using Silva taxonomic databases (Quast et al., 2013). QIIME software was used to generate species abundance information at different taxonomic levels. The bacterial DNA sequences were stored in the SRA of the NCBI database under the SRA accession PRJNA735314, BioSample accession SAMN19575439-19575483.

### Statistical Analyses

Differences in physicochemical parameters, biochar, and microbiological inoculum were subjected to analysis of variance. The difference between any two observed data was compared using Duncan’s tests with R 3.3.2 (R Development Core Team, 2015) at a statistical significance of *P* < 0.05.

Alpha diversity metrics, including ACE, Chao1, Shannon index, and Simpson diversity index, were calculated using the Mothur software. Differences between the different groups were examined using one-way analysis of variance. The difference between any two observed data was compared using Duncan’s test with statistical significance at *P* < 0.05. Pearson correlation coefficients were determined to evaluate the relationships between microbial alpha diversity and physicochemical properties. Statistical significance was set at *P* < 0.05. These analyses were performed using R 3.3.2 (R Development Core Team, 2015).

Relative bacterial abundance was determined and community structures were visualized using non-metric multi-dimensional scaling ordinations; linear discriminant analysis and effect size (LEfSe) analysis were performed as described by Sun et al. (2020). Analysis of similarities based on the Bray–Curtis dissimilarity matrices was conducted using the vegan package (Oksanen et al., 2013) in R 3.3.2. Bacterial taxa were significantly differentiated among the three different treatments using the randomForest package in R 3.3.2.

Mantel tests were performed to explore the relationship between bacterial community structure and physicochemical variables using the vegan package (Oksanen et al., 2013). Pearson correlation and redundancy analysis (RDA) were employed to evaluate relationships between bacterial community structure and physicochemical variables, as described by Sun et al. (2020). Independent effects of physicochemical variables were evaluated by r-squared values using rdacca.hp packages (Lai et al., 2021) of R 3.3.2. Important variables associated with physicochemical properties and bacterial taxa were ranked using the randomForest package of R 3.3.2.

## Results and Discussion

### Changes in Physicochemical Properties During the Composting Process With Different Treatments

**FIGURE 1:**
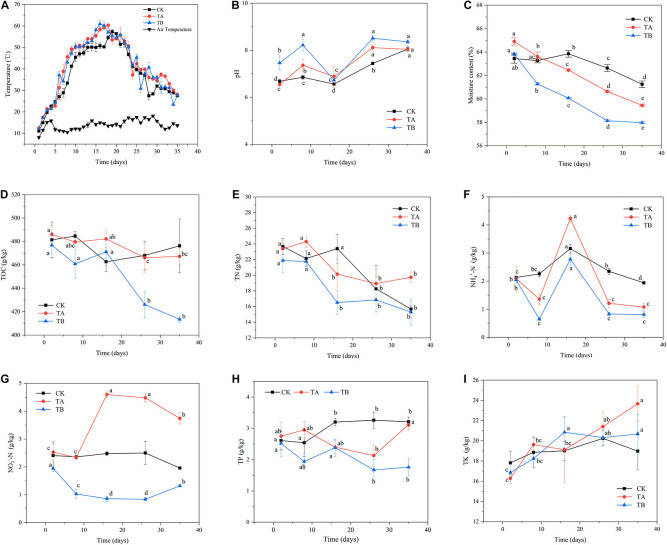
The changes in physicochemical properties during 35 days of aerobic composting with three treatments. **(A)** temperature, **(B)** pH, **(C)** moisture content, **(D)** total organic carbon, **(E)** total nitrogen, **(F)** nitrate nitrogen, **(G)** ammonium nitrogen, **(H)** total phosphorus, **(I)** total potassium. CK: without any treatment; TA-microbiological inoculum (microbiological inoculum volume/pile quality was 1%), TB-microbiological inoculum (1%) combined with 10% coconut shell biochar.

Changes in temperature are shown in Figure 1A. The results showed that TA and TB both accelerated the rate of temperature increase. The temperatures in TA and TB reached their peak (60°C) at 18 and 16 days, respectively, whereas CK reached its peak temperature (57°C) in 19 days. The temperature remained above 50°C for 14 days in TA and TB, and for 10 days in CK. Moreover, the temperature increases in TA and TB were greater than those in CK during the mesophilic phase. These results indicate that both microbial inoculation alone and integrated with biochar could promote organic matter degradation in the initial phase of composting and contribute considerable heat from microbes that ferment organic matter. Li C. et al. (2019) reported that the addition of a microbial inoculant could prolong the thermophilic stage by 2 days. Wang et al. (2019) indicated that the warming period was increased by 2 days in the treatment with *B. stearothermophilus*.

On the second day, the pH values for CK, TA, and TB were 6.68, 6.54, and 7.46, respectively (Figure 1B). These values were considered to influence the microbial activity during composting. Here, the pH range was 5.5–8.5. In this study, variation tendencies of the different treatments were similar. The pH value gradually increased during the first week, implying that ammonification was stronger than the production of organic acids from degradation of substrates. Wu et al. (2019) also found a similar trend in composting when phosphate was added. pH values significantly decreased until the thermophilic phase when they reached their lowest levels, which was mainly due to a peak in the volatilization of ammonia during the thermophilic phase (Agyarko-Mintah et al., 2017), and production of organic acids by intense decomposition of organic matter for thriving activities of microorganisms (Wei et al., 2018). Subsequently, pH values slowly increased during the cooling phase until they stabilized. TB displayed significantly higher alkalinity than TA and CK during most of the composting process, except on day 16. Duan et al. (2019b) reported that biochar treatment yielded higher alkalinity than treatment without biochar during the composting process.

The initial water contents for CK, TA, and TB were 63.44, 64.90, and 63.82%, respectively, and the water content of the TA sample was higher compared to that in other samples. The water content decreased continuously during composting, reaching 61.25, 59.45, and 59.97% on day 35, respectively (Figure 1C). The water content of CK was significantly higher than that of TA and TB (*P* < 0.05) (Table 1). A closed reactor might have led to insufficient water evaporation. Wang et al. (2019) showed that the water content ranged from 58.23 to 60.40% after composting using 19-L reactor bins.

**TABLE 1:** Physicochemical characteristics of the aerobic composting process for the three treatments.

**Sample ID**	**pH**	**Moisture content (%)**	**EC (mS/cm)**	**TOC (g/kg)**	**TN (g/kg)**	**NO^–^_3_-N (g/Kg)**	**NH_4_^+^-N (g/Kg)**	**TK (g/Kg)**	**TP (g/Kg)**
CK 2	6.68 ± 0.09a	63.44 ± 0.39a	3.33 ± 0.22	481.38 ± 15.22	23.67 ± 1.03	2.41 ± 0.49	2.13 ± 0.03	17.82 ± 1.16	2.61 ± 0.29
TA 2	6.54 ± 0.01a	64.90 ± 0.31b	3.36 ± 0.16	486.06 ± 7.36	23.39 ± 0.62	2.53 ± 0.29	2.09 ± 0.44	16.29 ± 0.26	2.75 ± 0.45
TB 2	7.46 ± 0.30b	63.82 ± 0.11a	3.08 ± 0.15	476.79 ± 10.78	21.88 ± 1.51	1.95 ± 0.11	2.04 ± 0.11	16.87 ± 1.05	2.52 ± 0.43
CK 8	6.85 ± 0.09a	63.27 ± 0.18a	3.18 ± 0.56	484.63 ± 3.77a	22.13 ± 0.98a	2.36 ± 0.06a	2.26 ± 0.09a	18.86 ± 1.53	2.54 ± 0.44ab
TA 8	7.36 ± 0.26b	63.62 ± 0.38a	3.37 ± 0.21	479.52 ± 1.22a	24.30 ± 0.72b	2.34 ± 0.08a	1.36 ± 0.18b	19.62 ± 0.63	2.95 ± 0.26b
TB 8	8.22 ± 0.25c	61.27 ± 0.09b	3.39 ± 0.23	460.8 ± 12.20b	21.76 ± 0.45a	1.03 ± 0.16b	0.65 ± 0.05c	18.24 ± 0.42	1.94 ± 0.25a
CK 16	6.56 ± 0.05	63.85 ± 0.29a	4.66 ± 0.47b	462.65 ± 8.48a	23.40 ± 1.85a	2.48 ± 0.07a	3.16 ± 0.15a	19.01 ± 0.96	3.20 ± 0.12a
TA 16	6.88 ± 0.45	62.46 ± 0.09b	5.40 ± 0.17c	482.20 ± 7.59b	20.14 ± 1.78a	4.60 ± 0.13b	4.23 ± 0.10b	19.12 ± 3.23	2.40 ± 0.22b
TB 16	6.73 ± 0.00	60.07 ± 0.04c	3.80 ± 0.17a	471.71 ± 11.59ab	16.51 ± 1.50b	0.86 ± 0.09c	2.78 ± 0.20c	20.85 ± 1.54	2.38 ± 0.28b
CK 26	7.44 ± 0.02a	62.63 ± 0.30a	3.49 ± 0.30	468.0 ± 12.15a	18.27 ± 2.96	2.50 ± 0.42a	2.35 ± 0.11a	20.24 ± 0.74	3.26 ± 0.26a
TA 26	8.11 ± 0.14b	60.64 ± 0.06b	3.65 ± 0.34	466.1 ± 12.38a	18.93 ± 0.86	4.48 ± 0.14b	1.21 ± 0.15b	21.40 ± 1.48	2.12 ± 0.34b
TB 26	8.51 ± 0.12c	58.14 ± 0.07c	3.34 ± 0.03	426.04 ± 11.06b	16.85 ± 1.54	0.84 ± 0.05c	0.83 ± 0.05c	20.32 ± 0.79	1.67 ± 0.33b
CK35	8.05 ± 0.10a	61.25 ± 0.28a	3.77 ± 0.25	476.2 ± 23.00a	15.67 ± 0.38a	1.96 ± 0.03a	1.94 ± 0.02a	18.98 ± 1.87a	3.21 ± 0.15a
TA 35	8.04 ± 0.03a	59.45 ± 0.11b	3.65 ± 0.34	467.24 ± 7.22a	19.70 ± 0.67b	3.74 ± 0.20b	1.08 ± 0.12b	23.67 ± 17.08b	3.12 ± 0.17a
TB 35	8.36 ± 0.07b	59.97 ± 0.08c	3.48 ± 0.12	413.47 ± 2.68b	15.29 ± 1.67a	1.32 ± 0.04c	0.80 ± 0.12c	20.67 ± 1.97ab	1.76 ± 0.28b

*Values represent means ± standard deviation. CK, aerobic compost with no additive. TA, aerobic compost with microbiological inoculation. TB, aerobic compost with microbiological inoculum combined with 10% biochar.*

*TN, total nitrogen; TOC, total organic carbon; C/N, the ratio of TC to TN; NO_3_^–^-N, and NH_4_^+^-N, nitrate nitrogen and ammonium nitrogen; TP, total phosphorus; TK, total potassium; EC, electrical conductivity.*

*Different letter indicate significant correlations (P < 0.05).*

At the initial stage (on day 2), the TOC concentrations in CK, TA, and TB were 481.38, 486.06, and 476.79 g/kg, respectively (Figure 1D). The TOC concentration did not vary significantly in CK and TA at different stages. A high TOC concentration is attributed to a low degree of decomposition due to the high recalcitrant lignin content (Khan et al., 2014). The TOC content sharply decreased to 413.47 g/kg after 16 days in TB at the final stage (Figure 1D). Furthermore, the TOC content in TB was significantly lower than that in CK and TA during the composting process (Table 1). This indicates that microbiological inoculation integrated with biochar can accelerate the decomposition of recalcitrant lignin.

At the initial stage (on day 2), TN contents of CK, TA, and TB were 23.67, 23.39, and 21.88 g/kg, respectively (Figure 1E). The TN content showed a general downward trend, which was demonstrated by the continuation of fermentation and explained by the volatilization of nitrogen in the form of ammonia during the temperature-rising stage (Zhao et al., 2016). Finally, the TN content of TA was significantly higher than CK and TB (*P* < 0.05) (Table 1). The total N loss was 15.77% in TA, whereas it was 33.80 and 30.11% in CK and TB, respectively, demonstrating microbiological inoculation alone could be crucial for reserving nitrogen.

NH_4_^+^-N and NO_3_^–^-N are the main forms of inorganic nitrogen in the compost and reflect nitrogen transformations. In all three treatments, the NH_4_^+^-N contents were at a relatively high level on day 2 at 2.13, 2.09, and 2.04 g/kg, respectively. The contents increased drastically, reaching their maximum values on day 16, to 3.16, 4.23, and 2.78 g/kg, respectively (Table 1). This indicated that mineralization and ammonification of organic nitrogen compounds was intense at this stage. However, nitrification is limited to some extent at high temperatures (Yu et al., 2020). It is worth noting that the NH_4_^+^-N content of TA was significantly higher than that of TB and CK on day 16, reaching 4.23 g/kg (*P* < 0.05) (Table 1). This implied that the degree of ammonification and organic nitrogen hydrolysis were promoted under microbiological inoculation treatment and lower nitrification. After reaching the maximum values, the NH_4_^+^-N concentration profiles decreased gradually and stabilized at the end of the composting process (Figure 1F). At this stage, a large amount of NH_3_ was volatilized, and the recovery of nitrification caused the NH_4_^+^-N concentration to decrease after the thermophilic phase. During the cooling stage, the NH_4_^+^-N content of TA and TB sharply declined compared to that of CK, probably because of more activities by microorganisms, including the formation of NO_3_^–^-N (Jiang et al., 2015). The variation tendency of NH_4_^+^-N during composting was consistent with the findings of Ravindran et al. (2019). Across the entire composting period, the NO_3_^–^-N content remained stable in CK and decreased continuously in the mesophilic and thermophilic stages in TB. Then, the NO_3_^–^-N content significantly increased during the last maturity stage in TB due to nitrification (*P* < 0.05). The NO_3_^–^-N concentration was significantly lower in CK and TB than in TA after day 8 (Table 1) and surged significantly (*P* < 0.05) from days 8 to 26 in TA across the warming and thermophilic phases. The NO_3_^–^-N concentration increased from 2.53 to 4.48 g/kg in TA (Figure 1G). Previous studies have shown that stronger nitrification leads to the accumulation of NO_3_^–^-N (Sun et al., 2019; Wan et al., 2020). Biological nitrification involves two steps: first, oxidizing NH_4_^+^-N to NO_2_^–^-N, and further oxidizing NO_2_^–^-N to NO_3_^–^-N (Zhong et al., 2020). The reduction of NO_3_^–^-N was found on day 35 in TA and CK. This was because NO_3_^–^ and NO_2_^–^ shifted to NO, N_2_O through denitrification (Zhong et al., 2020), and assimilation which promoted the generation of organic nitrogen. It is worth noting that ammonia oxidation occurs during the maturation phase of composting because nitrifying bacteria have limited survival at temperatures above 40°C (Wan et al., 2020). Other reports have not found that the increase in NO_3_^–^-N occurs at temperatures above 40°C or mainly occurs in the cooling stage when applied with microbiological incubation (Li J. et al., 2019; Yu et al., 2020). In our study, the thermophilic microbial incubation treatment showed stable conversion of NH_4_^+^-N to NO_3_^–^-N at temperatures above 40°C, indicating that microorganisms can be resistant to high temperatures and capable of nitrifying.

The TP ranges were 2.61–3.21, 2.75–3.12, and 1.67–2.52 g/kg in CK, TA, and TB, respectively (Figure 1H). This result indicated that microbial inoculation integrated with biochar could accelerate the decomposition of TP. Furthermore, TK had an increasing trend in TA and TB, and there were no significant changes in CK (Figure 1I).

GI is regarded as an acceptable index to evaluate compost maturity. GI was over 50%, indicating that the compost was not phytotoxic. On the second day of composting, GI values were 17.78, 24.18, and 24.32% in CK, TA, and TB, respectively (Supplementary Figure 1). The GI value gradually increased with the compost processing, and when the composting ended, GI values reached 56.31, 73.57, and 62.64% in CK, TA, and TB, respectively (Supplementary Figure 1).

### Microbiological Alpha Diversity Analysis

**FIGURE 2:**
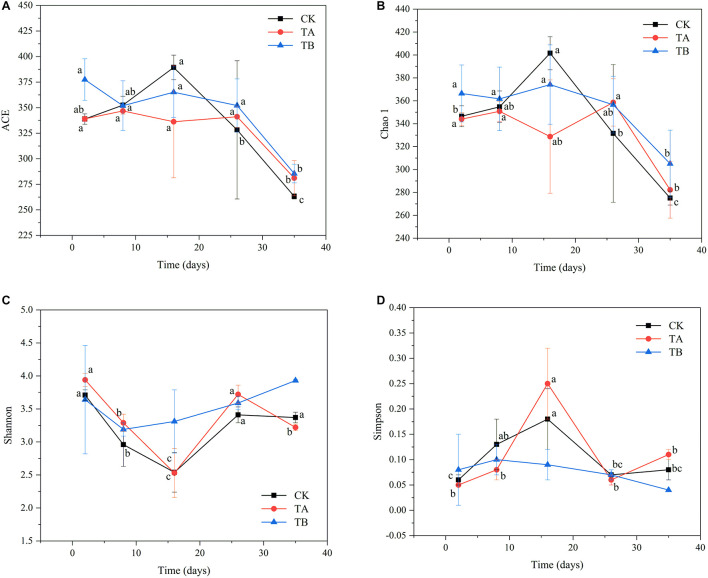
The changes of alpha diversity of the bacterial community during 35 days of aerobic composting. **(A)** ACE index; **(B)** Chao1 index; **(C)** Shannon index; **(D)** Simpson index.

In total, 3,467,510 clean bacterial tags and 4,464 OTUs were obtained from 45 samples. The numbers of OTUs in CK, TA, and TB were 1,473, 1,486, and 1,505, respectively (Supplementary Table 3). There were no significant differences in the OTUs among composting treatments (*P* > 0.05). The OTU confines in this study were similar to those observed for the same treatments in cow manure composting (270–572) (Duan et al., 2019a), which was much lower than those observed during PM composting with rice straw (1,372–2,175) (Zhou et al., 2019). Consistent with the results of previous studies, the number of OTUs increased during the mesophilic phase and declined during the mature phase of composting (Ma et al., 2018). All rarefaction curves approached a plateau, suggesting that the sequencing depth for all samples sufficed to cover the bacterial community diversity (Supplementary Figure 2). For alpha diversity, ACE and Chao1 reflected the variation in microbiological richness, and Shannon and Simpson indices reflected the microbiological diversity. During aerobic composting, bacterial richness decreased significantly after the thermophilic phase (16 d) in CK (Figure 2). However, bacterial richness remained stable in TA and TB (Figure 2). At the end of composting, TB had the highest ACE (288.51) and Chao1 index (305.04) (Supplementary Table 3). The bacterial diversity of CK and TA decreased to their lowest levels in the thermophilic phase (16 d) and then increased during the cooling stage (26 days). In contrast, bacterial diversity remained stable during aerobic composting in TB (Supplementary Table 3 and Figure 2). Notably, TB treatment yielded the highest bacterial diversity (with Shannon and Simpson indices of 3.93 and 0.044, respectively), followed by TA, then CK, which had the lowest bacterial diversity (*P* < 0.05) (Supplementary Table 3 and Figure 2). Overall, the richness and diversity of bacterial communities thrived upon interaction with the bacterial consortium integrated with biochar.

Pearson correlation coefficients indicated that pH, water content, EC, and TN were positively correlated with bacterial ACE (*P* = 0.019, 0.08, 0.036, and 0.014, respectively) (Supplementary Figure 4), whereas TK was negatively correlated with bacterial ACE (*P* = 0.002) (Supplementary Figure 4). The pH level, water content, and TN were positively correlated with bacterial Chao1 (*P* = 0.017, 0.036, and 0.027, respectively) (Supplementary Figure 4). EC, NO_3_^–^-N, and NH_4_^+^-N were positively correlated with the bacterial Shannon index (*P* < 0.001, *P* = 0.006, and *P <* 0.001, respectively) (Supplementary Figure 4). EC and NH_4_^+^-N were negatively correlated with the bacterial Simpson index (*P* = 0.002 and *P <* 0.001, respectively) (Supplementary Figure 4).

### Changes in Bacterial Community Structure

**FIGURE 3:**
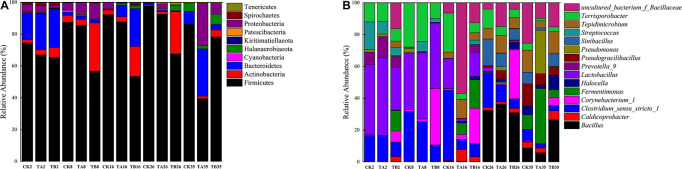
Community composition of bacteria at the taxonomic levels of phylum **(A)** and genus **(B)**.

In total, 10 phyla, 18 classes, 42 orders, 86 families, and 208 genera of bacteria were detected in the samples. According to assignment results at the phylum level, *Firmicutes* was the most abundant phylum across all samples (39.36–97.60%). *Bacteroidetes*, *Actinobacteria*, and *Proteobacteria* were the three dominant phyla (Figure 3 and Supplementary Table 4). These bacteria have been widely found in compost (Ventorino et al., 2015; Wang et al., 2020). Moreover, previous studies have revealed that the dominant phyla in PM compost were *Proteobacteria*, *Actinobacteria*, and *Firmicutes* (Li C. et al., 2019) and physicochemical properties and additional dosages have been shown to affect the bacterial community during composting (Awasthi et al., 2017c).

*Firmicutes* play an important role in the degradation of lignocellulose substrates during the entire composting process (Duan et al., 2019a). *Firmicutes* also exhibit tolerance to heat during composting (Yi et al., 2012). Li J. et al. (2019) reported that the relative abundance (RAs) of *Firmicutes* decreased for all biochar addition treatments, which was also observed in this study. The relative abundance of *Bacteroidetes* was higher on day 2 of this study. *Bacteroidetes* have been shown to disintegrate cellulose into brief fatty acids (Wang et al., 2018). In this study, at the mesophilic stage, *Bacteroidetes* showed a decreasing trend in all three treatments, and *Actinobacteria* increased markedly in TB (30.4%) (Figure 3 and Supplementary Table 4). With increasing temperature, the abundance of *Bacteroidetes* continued to decrease in CK, whereas it increased in TA and TB. *Actinobacteria* had a relatively stable abundance in TB until day 26. *Actinobacteria* have a vital effect on the degradation of obstinate cellulose and lignin (Duan et al., 2019a). *Actinobacteria* and *Bacteroidetes* were higher in TA and TB than in CK, which resulted from TA and TB both increasing the temperature of the composting piles. Since they are thermo-tolerant, they can be active and survive at high temperatures and in the rest phases of composting (Sun et al., 2019). The higher RAs of these two phyla implied that the bacterial consortium alone or integrated with biochar could effectively boost cellulose degradation. At the end of composting, *Actinobacteria* (29.78%) and *Proteobacteria* (26.79%) were relatively enriched in TA (Figure 3 and Supplementary Table 4).

The most abundant 15 genera were *Lactobacillus, Bacillus, Caldicoprobacter, Clostridium sensu stricto, Corynebacterium, Fermentimonas, Halocella, Prevotella, Pseudomonas, Streptococcus, Terrisporobacter, Sinibacillus, Tepidimicrobium, Pseudogracilibacillus*, and uncultured *Bacillaceae*. The relative abundance changed significantly with the composting process. On days 2–8 of composting, all treatments yielded a significant higher proportion of *Lactobacillus* (26.88–46.71%). *Lactobacillus* is a mesophilic organic acid-producing bacterium that often appears at the beginning of the composting process (Partanen et al., 2010). The secondary genera were *Clostridium_sensu_stricto_1* (9.03–31.69%), *Terrisporobacter* (7.82–27.83%), *Streptococcus* (0.67–17.27%), and *Prevotella_9* (6.89∼13.41%) (Figure 3 and Supplementary Table 5). *Clostridium _sensu_stricto_1* strains are reported to be distributed in composts and animal gastrointestinal tracts (Yin et al., 2017) and have been shown to mainly inhabit the pig gastrointestinal tract ecosystem, and have also been observed in fresh PM (Snell-Castro et al., 2005; Huang et al., 2018). Notably, TB contained higher numbers of *Fermentimonas* (12.85%) on day 8 and *Corynebacterium* during days 2–8 (7.05–35.51%) (Figure 3 and Supplementary Table 5). Therefore, *Clostridium*, *Terrisporobacter*, *Corynebacterium*, *Pseudomonas*, and *Prevotella_*9 were dominant in PM composting (Ma et al., 2018; Duan et al., 2019b).

On day 16, the dominant genus showed no significant changes in CK; uncultured *Bacillaceae* was the dominant genus (56.00%) in TA, followed by *Tepidimicrobium* (12.07%) (Figure 3 and Supplementary Table 5). *Bacillaceae* are carbon-degrading microbes that have been shown to degrade cellulose during composting (Meng et al., 2018). *Tepidimicrobium* was found when the pile temperature varied from mesophilic to thermophilic during kitchen waste composting, suggesting that this genus could be selectively enriched under thermophilic conditions (Jiang et al., 2020). Furthermore, *Tepidimicrobium* has previously been regarded as a highly degradable microbial species of cellulolytic compounds (Liu et al., 2020). *Fermentimonas* (7.08%), *Caldicoprobacter* (7.33%), and *Clostridium_sensu_stricto*_1 (6.42%) accounted for a considerable proportion of TA (Figure 3 and Supplementary Table 5). *Fermentimonas* has been isolated from biogas-producing reactors (Maus et al., 2017). *Caldicoprobacter* is a thermophilic, xylanolytic bacterium that is insolated in oil palm trunk compost (Widyasti et al., 2018). *Clostridium* spp. have been reported to have thermal resistance (Dharmasena et al., 2019) and could use cellulose (Reed et al., 2014). *Clostridium_sensu_stricto_1* was the chief component in the mesophilic and thermophilic phases during swine manure composting (Yin et al., 2017). Notably, TB had a more abundant bacterial community than CK and TA at the thermophilic stage, as biochar treatment improved the porosity and degradation rate of organic waste and therefore, the diversity of bacteria in the compost was higher. Besides the same genus as TA, *Fermentimonas* (18.03%) and *Corynebacterium* (22.22%) continued to exist in TB at this stage (Figure 3 and Supplementary Table 5).

At day 26, *Bacillus* (30.90–36.19%) became predominant in all three treatments, indicating its importance (Figure 3 and Supplementary Table 5). The *Bacillus* genus has also been shown to be related to organic matter biodegradation and organic matter-degrading bacteria (Yi et al., 2012). Partanen et al. (2010) found that the unloading end of composting plants contained a large proportion of *Bacillus* sequences. Yi et al. (2012) observed that culturable *Bacillus* species in middle-level composting during the temperature-rising and cooling stages were more diverse than those from the other layers. *Clostridium_sensu_stricto_*1 (6.02–21.19%), *Tepidimicrobium* (6.74–10.94%), and *Terrisporobacter* (5.37–11.98%) were still maintained at a considerable abundance. At this stage, *Sinibacillus* occupied 6.94–16.74% (Figure 3 and Supplementary Table 5). As a thermo-tolerant genus of the *Bacillaceae* family, *Sinibacillus* possesses a strong tolerance to radiation, chemicals, heat, and drought. *Sinibacillus* has been reported to be dominant in wheat straw, chicken manure compost, and mushroom dreg and chicken manure compost in the thermophilic phase (Kong et al., 2020). *Corynebacterium* accounted for 30.79% of TB, which was reflected in the vigorous cellulose degradation (Figure 3 and Supplementary Table 5).

**FIGURE 4:**
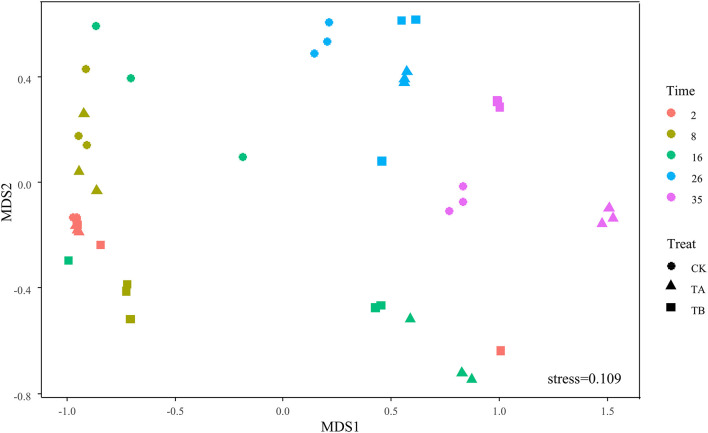
Non-metric multidimensional scaling (NMDS) biplot of Bray–Curtis dissimilarity matrix of bacteria at the genera level (*R*^2^ = 0.988).

**FIGURE 5:**
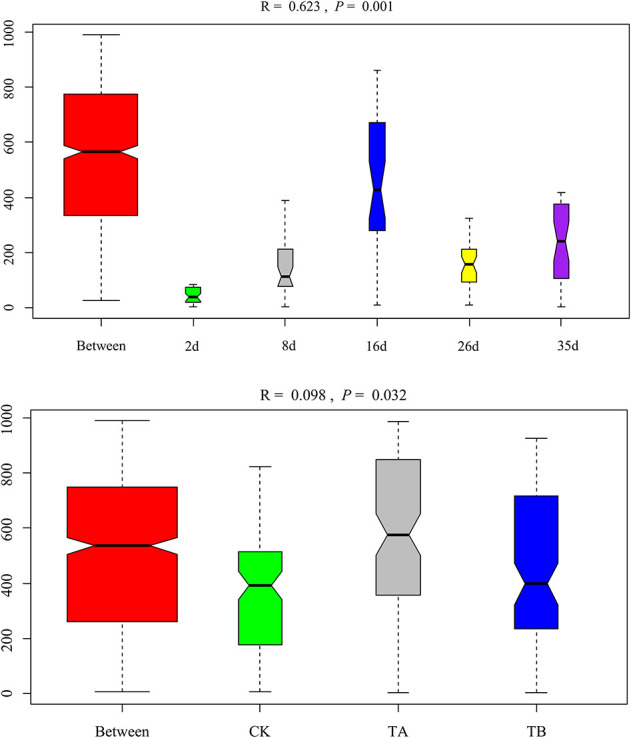
Analysis of similarities (ANOSIM) plot of Bray–Curtis dissimilarity ranks between composting period and treatment for bacterial composition at the genus level.

On day 35, *Fermentimonas*, *Pseudomonas*, *Pseudogracilibacillus*, *Tepidimicrobium*, *uncultured Bacillaceae*, *Clostridium_sensu_stricto*, and *Bacillus* were the dominant genera; however, their relative abundances were significantly different among the three treatments. Dissimilarities in bacterial community composition across the samples were identified using the non-metric multi-dimensional scaling biplot (Figure 4). This study revealed that the bacterial community structure varied significantly among composting phases. This indicated that composting materials influenced the physicochemical conditions during composting, which influenced the microbial structure and metabolism (Bello et al., 2020), like the observations of Meng et al. (2019). Notably, the three treatments had similar community structures on day 2 and different community structures after their separate treatments on day 35 (Figure 4), implying that the treatments influenced bacterial community composition. Interestingly, analysis of similarities further revealed that the composting period and treatment influenced the bacterial community composition at the genus level. The community dissimilarity matrix was significantly affected by the composting period (*R*^2^ = 0.623, *P* = 0.001) and the treatments (CK, TA, and TB) (*R*^2^ = 0.098, *P* = 0.032) (Figure 5).

**FIGURE 6:**
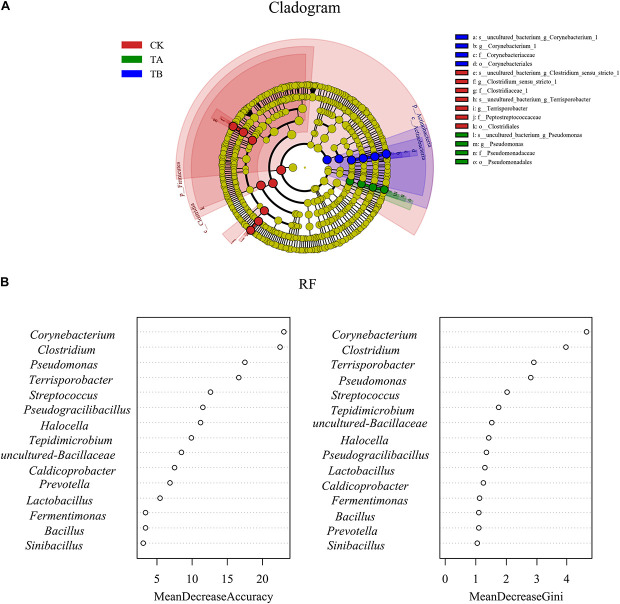
**(A)** Bacterial taxa significantly differentiated between three different treatments identified by linear discriminant analysis effect size (LEfSe) using the default parameters. Bacterial taxa that were differentially abundant in different groups visualized in a cladogram. **(B)** Bacterial taxa significantly differentiated between the three different treatments using random forest.

**FIGURE 7:**
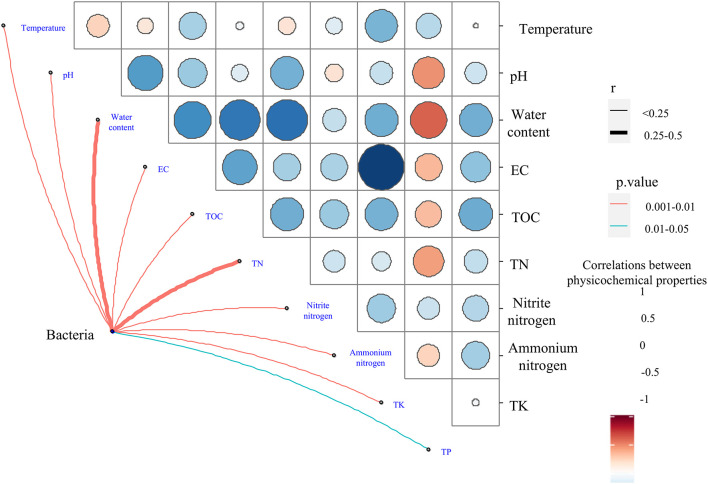
Relationships between bacterial community compositions at the genus level and physicochemical variables revealed by the Mantel test. Line width corresponds to the partial Mantel’s r statistic, and line color denotes the statistical significance based on 999 permutations.

To determine the functional community/communities in the different treatments, LEfSe was conducted to identify the groups that displayed significant differences across treatments; the identified indicator groups are shown in Figure 7. The phylum *Firmicutes* was significantly enriched in the CK. There were three significantly abundant bacterial taxa, including *Clostridiaceae*, *Terrisporobacter*, and *Peptostreptococcaceae*. *Pseudomonas* belonging to *Pseudomonadaceae* and the *Proteobacteria* phylum was the most abundant in TA. *Corynebacterium* belonging to the *Corynebacteriaceae* and the *Actinobacteria* phylum was overrepresented in TB (Figure 7A), implying a more thorough degradation. Like the results of LEfSe, the random forest revealed that *Corynebacterium*, *Clostridium*, *Pseudomonas*, and *Terrisporobacter* were significantly different among the three different treatments (Figure 7B).

### Association of Bacterial Composition With Physicochemical Properties During the Composting Process

**FIGURE 8:**
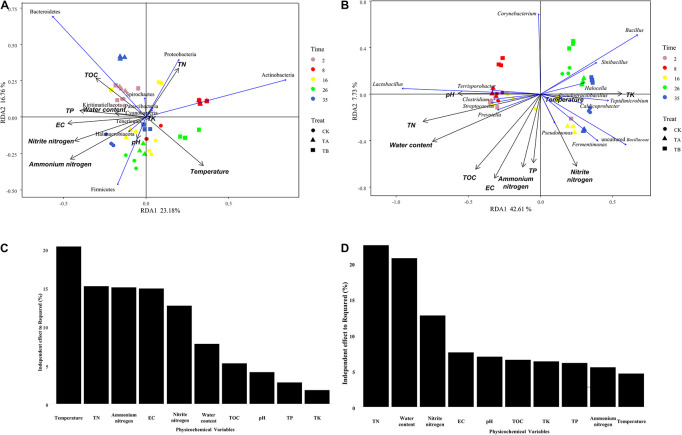
Bi-plot from the redundancy analysis (RDA) showing the relationships between phylum-level **(A)** and genus-level **(B)** of bacterial community compositions and physicochemical variables for all samples. Independent effect of physicochemical variables on bacterial community composition at the phylum level **(C)** and genus level **(D)**.

Mantel tests indicated that all 10 physicochemical properties (i.e., temperature, pH, water content, EC, TOC, TN, nitrite nitrogen, ammonium nitrogen, TK, and TP) significantly influenced the bacterial community composition (*P* < 0.05) (Figure 8 and Supplementary Table 6). Among them, TN and water content had *r*-values of 0.418 and 0.412, respectively. Furthermore, all 10 physicochemical properties were found to be correlated with the bacterial communities at the phylum and genus levels by RDA. At the phylum level, axis 1 interpreted 23.18% of the variance, and 16.76% of the variance was interpreted by axis 2 (Figure 6A). Temperature had an independent effect on the highest proportion (20.34%), followed by TN and ammonium nitrogen, accounting for 15.22 and 15.07%, respectively (Figure 6C). TA and TB both increased the temperature of the composting piles, influencing the community structure. *Firmicutes* and *Actinobacteria* showed a close positive relationship with temperature, and *Actinobacteria* was relatively abundant in TA and TB on day 8. *Firmicutes* have a wide range of adaptations and can dominate the composting process. However, *Proteobacteria* were the main microorganisms in the metaphase and mature stages of composting, and *Bacteroidetes* tolerate high levels, as the composting piles gradually cool, which is more favorable for *Bacteroidetes*. As an important nutrient resource for microbes, TN was negatively correlated with *Firmicutes* and positively correlated with *Actinobacteria* and *Proteobacteria*. Therefore, TN content in TA was significantly higher than others, and the result of RDA showed *Proteobacteria* was relatively abundant in TA on day 35. Ammonium nitrogen showed the opposite trend (Figure 6A). CK on day 35 had the highest NH_4_^+^-N content and relatively abundant *Firmicutes.* Some members of *Firmicutes* have been reported to dominate ammonia production (Yabu et al., 2011).

At the genus level, 42.61% of the variance was interpreted by axis 1, and axis 2 interpreted 7.73% of the variance (Figure 6B). TN, water content, and nitrite nitrogen had the most significant influence on the bacterial community, with independent effect r-squared values of 22.52, 20.76, and 12.76%, respectively (Figure 6D). Bacterial consortium addition increased the total N and nitrite nitrogen content, and biochar combined with microbial inoculation increased the pH and decreased the water content, which could influence the bacterial community composition during composting. Microflora should acquire N nutrients from the composting process to support their growth and reproduction (Li et al., 2017). The quality and quantity of substrate supply, such as the N source, could affect the bacterial communities. Therefore, it was reasonable to observe a strong link between the N content and bacterial community composition. Zhou et al. (2019) reported similar relationships. Meanwhile, TN was closely correlated with *Lactobacillus*, *Bacillus*, *Caldicoprobacter*, *Clostridium sensu strict*, *Halocella*, *Streptococcus*, *Terrisporobacter*, *Sinibacillus*, *Tepidimicrobium*, and *Pseudogracilibacillus* (Figure 6B and Supplementary Figure 5). Furthermore, the relationships between the two dominant genera across all treatments, *Lactobacillus* and *Bacillus*, and four genera as the indicator groups in CK, TA, and TB, which were *Clostridium*, *Terrisporobacter*, *Pseudomonas*, and *Corynebacterium*, were explored using random forest. *Bacillus* was significantly influenced by EC, TN, TOC, and water content (Supplementary Figure 6). *Bacillus* plays an important role in the cycling of carbon and nitrogen. *Bacillus* species have also been shown to have a strong ability to decompose organic films and degrade nitrites (Yi et al., 2012). TN, pH, and water content were the driving forces for *Lactobacillus*, the organic acid-producing taxa (Supplementary Figure 6). *Clostridium* and *Terrisporobacter* were significantly differentiated in the CK and were closely associated with TN (Supplementary Figure 6). *Pseudomonas* was significantly differentiated in TA and positively associated with nitrite nitrogen and TK (Supplementary Figure 6). *Pseudomonas* was higher in TA than in CK, which is consistent with the results of Duan et al. (2019a) in cow manure. *Pseudomonas spp.* have also been shown to have efficient heterotrophic aerobic nitrification capability (Deng et al., 2021; Wei et al., 2021). TA had the maximum TN and TK at the end of composting, thereby demonstrating more efficient compost quality after interaction with the bacterial consortium during composting. *Corynebacterium* was enriched in TB and was closely associated with the pH level (Supplementary Figure 6). *Corynebacterium* played a vital role in degradation, leading to a more thorough degradation of organic matter in TA and TB than in CK. *Corynebacterium* also accounted for a relatively higher proportion of TB, thus explaining the lowest TOC content observed in TB.

## Conclusion

This study demonstrated that thermophilic microbial inoculation alone and combined with biochar influences several physicochemical characteristics of PM composting with wheat straw. Compared with CK, the addition of microbial inoculation alone and combined with biochar accelerated the composting process. The most abundant phyla in descending order were *Firmicutes*, *Bacteroidetes*, *Actinobacteria*, and *Proteobacteria*. The relative abundance of genera changed significantly with the composting period and treatment. Microbial inoculation treatment significantly enhanced the total nitrogen content for enriching *Pseudomonas*, but significantly accelerated TOC degradation when interacting with biochar for higher richness and diversity as well as overrepresented *Corynebacterium*.

## Data Availability Statement

The original contributions presented in the study are included in the article/Supplementary Material, further inquiries can be directed to the corresponding author/s.

## Author Contributions

LS contributed to conception and design for the study, analysis of data, drafted, and revised the manuscript. JL, RW, YL, and ZW collected the materials and conducted the experiment. ML and DT coordinated to acquire the data. LM revised the manuscript. All authors reviewed and approved the final manuscript.

## Conflict of Interest

ML was employed by company Lanzhou Zhuangyuan Pasture Company Limited. The remaining authors declare that the research was conducted in the absence of any commercial or financial relationships that could be construed as a potential conflict of interest.

## Publisher’s Note

All claims expressed in this article are solely those of the authors and do not necessarily represent those of their affiliated organizations, or those of the publisher, the editors and the reviewers. Any product that may be evaluated in this article, or claim that may be made by its manufacturer, is not guaranteed or endorsed by the publisher.
