# Supplementary material for: Different Effects of Thermophilic Microbiological Inoculation With and Without Biochar on Physicochemical Characteristics and Bacterial Communities in Pig Manure Composting
Source: Front Microbiol. 2021 Nov 16;12:746718. doi: 10.3389/fmicb.2021.746718 (PMC8660119; doi:10.3389/fmicb.2021.746718)
Supplement: Supplementary file 1 [file Data_Sheet_1.docx]

**Table S1.** The physicochemical characteristics of the raw materials.

|  | Pig mature | Wheat straw | Biochar |
| --- | --- | --- | --- |
| Total carbon (g/kg) | 220.52 | 481.88 | 252.24 |
| Total nitrogen (g/kg) | 13.63 | 11.49 | - |
| C/N | 16.18 | 41.93 | - |
| Water content (%) | 63.77 | - | 5.0 |
| pH | 8.33 | - | 7.2 |
| Total [phosphorus](javascript:;) (g/kg) | 3.56 | 0.62 | - |
| Total potassium (g/kg) | 13.12 | 22.43 | 10.46 |

**Table S2.** The DNA concentration of each sample measured by fluorometric quantification.

| Sample ID | Concentration (ng/μL) | Sample ID | Concentration (ng/μL) | Sample ID | Concentration (ng/μL) |
| --- | --- | --- | --- | --- | --- |
| CK 2a | 19.3 | TA 2a | 25.6 | TB 2a | 23.5 |
| CK 2b | 22.8 | TA 2b | 38.6 | TB 2b | 28.1 |
| CK 2c | 15.7 | TA 2c | 29.5 | TB 2c | 21.6 |
| CK 8a | 7.1 | TA 8a | 8.1 | TB 8a | 7.5 |
| CK 8b | 5.7 | TA 8b | 4.7 | TB 8b | 6.0 |
| CK 8c | 4.3 | TA 8c | 9.3 | TB 8c | 9.1 |
| CK16a | 22.1 | TA 16a | 2.0 | TB 16a | 31.9 |
| CK 16b | 17.1 | TA 16b | 16.9 | TB 16b | 25.6 |
| CK 16c | 6.6 | TA 16c | 0.2 | TB 16c | 7.5 |
| CK 26a | 50.9 | TA 26a | 18.7 | TB 26a | 35.5 |
| CK 26b | 32.8 | TA 26b | 35.3 | TB 26b | 31.0 |
| CK 26c | 0.7 | TA 26c | 33.2 | TB 26c | 45.6 |
| CK 35a | 37.6 | TA 35a | 47.4 | TB 35a | 36.3 |
| CK 35b | 29.3 | TA 35b | 44.1 | TB 35b | 36.8 |
| CK35c | 47.8 | TA 35c | 53.1 | TB 35c | 38.1 |

CK, aerobic compost with no additive. TA, aerobic compost with microbiological inoculation. TB, aerobic compost with microbiological inoculum combined with 10% biochar.

**Table S3** Illumina sequencing reads and alpha diversity of bacterial community.

| Sample ID | Valid tags | OTUs | ACE | Chao1 | Shannon index | Simpson index |
| --- | --- | --- | --- | --- | --- | --- |
| CK 2 | 61181±197 | 331±3 | 338.85±5.15^a^ | 346.51±9.19^a^ | 3.71±0.08^a^ | 0.064±0.005^a^ |
| TA 2 | 62818±3577 | 332±2 | 339.04±2.50^a^ | 344.01±5.77^a^ | 3.94±0.10^a^ | 0.048±0.004^a^ |
| TB 2 | 64081±2292 | 325±59 | 377.44±20.39^b^ | 366.38±24.87^a^ | 3.64±0.82^a^ | 0.080±0.067^a^ |
| CK 8 | 66558±2072 | 323±10 | 382.46±8.63^a^ | 354.71±13.87^a^ | 2.94±0.33^a^ | 0.132±0.051^a^ |
| TA 8 | 62003±2303 | 326±4 | 346.78±3.03^a^ | 350.79±8.62^a^ | 3.29±0.13^a^ | 0.082±0.021^a^ |
| TB 8 | 62722±174 | 329±12 | 351.92±24.37^a^ | 361.69±27.71^a^ | 3.19±0.10^a^ | 0.096±0.030^a^ |
| CK 16 | 64322±3200 | 310±11 | 389.35±12.00^a^ | 401.56±14.47^a^ | 2.54±0.30^a^ | 0.175±0.055^a^ |
| TA 16 | 66622±1112 | 285±48 | 336.25±54.83^a^ | 328.74±49.47^b^ | 2.53±0.37^a^ | 0.248±0.073^a^ |
| TB 16 | 61828±1015 | 302±53 | 365.12±24.75^a^ | 374.13±34.67^a^ | 3.31±0.48^c^ | 0.087±0.031^c^ |
| CK 26 | 65930±394 | 282±66 | 328.29±67.63^a^ | 331.52±60.17^a^ | 3.41±0.12^a^ | 0.066±0.004^a^ |
| TA 26 | 64216±1130 | 296±17 | 341.11±10.45^a^ | 358.57±20.69^a^ | 3.72±0.14^b^ | 0.060±0.005^a^ |
| TB 26 | 64080±2030 | 284±12 | 352.09±26.01^a^ | 356.55±24.71^a^ | 3.59±0.10^b^ | 0.070±0.014^a^ |
| CK35 | 66862±360 | 227±3 | 263.22±2.55^a^ | 275.06±6.09^a^ | 3.17±0.08^a^ | 0.107±0.019^a^ |
| TA 35 | 65504±158 | 247±11 | 281.10±17.11^a^ | 282.23±24.73^a^ | 3.22±0.05^b^ | 0.105±0.007^b^ |
| TB 35 | 66074±138 | 265±5 | 288.51±9.09^b^ | 305.04±29.30^a^ | 3.93±0.02^c^ | 0.044±0.002^c^ |

Values represent means ± standard deviation. Different letter indicate significant correlations (*P* < 0.05). CK, aerobic compost with no additive. TA, aerobic compost with microbiological inoculation. TB, aerobic compost with microbiological inoculum combined with 10% biochar.

**Table S4.** The relative abundances of top fifteen bacterial phyla of aerobic compost processing with three treatments.

| Sample ID | *Firmicutes* | *Actinobacteria* | *Bacteroidetes* | *Proteobacteria* | *Cyanobacteria* | *Halanaerobiaeota* | *Kiritimatiellaeota* | *Patescibacteria* | *Spirochaetes* | *Tenericutes* |
| --- | --- | --- | --- | --- | --- | --- | --- | --- | --- | --- |
| CK2 | 74.47% | 1.99% | 16.61% | 4.69% | 0.47% | 0.00% | 0.10% | 0.34% | 1.15% | 0.18% |
| TA2 | 66.92% | 3.20% | 22.85% | 4.17% | 0.30% | 0.00% | 0.07% | 0.39% | 1.72% | 0.37% |
| TB2 | 65.34% | 6.29% | 24.14% | 1.80% | 0.25% | 0.20% | 0.02% | 0.23% | 1.54% | 0.20% |
| CK8 | 87.40% | 4.36% | 1.99% | 4.21% | 1.32% | 0.00% | 0.05% | 0.26% | 0.30% | 0.10% |
| TA8 | 85.56% | 3.70% | 2.12% | 7.27% | 0.54% | 0.00% | 0.06% | 0.42% | 0.23% | 0.09% |
| TB8 | 56.67% | 30.40% | 1.75% | 9.93% | 0.34% | 0.00% | 0.03% | 0.37% | 0.41% | 0.11% |
| CK16 | 92.21% | 2.90% | 0.29% | 4.08% | 0.33% | 0.06% | 0.00% | 0.04% | 0.03% | 0.05% |
| TA16 | 87.70% | 3.28% | 7.20% | 0.45% | 0.04% | 0.99% | 0.00% | 0.04% | 0.01% | 0.28% |
| TB16 | 53.41% | 18.79% | 23.67% | 0.84% | 0.11% | 1.60% | 0.02% | 0.15% | 0.99% | 0.42% |
| CK26 | 97.60% | 0.63% | 0.89% | 0.15% | 0.02% | 0.42% | 0.00% | 0.01% | 0.01% | 0.27% |
| TA26 | 92.42% | 1.74% | 0.78% | 3.05% | 0.02% | 0.95% | 0.00% | 0.02% | 0.00% | 1.02% |
| TB26 | 67.53% | 26.53% | 0.62% | 0.25% | 0.02% | 3.38% | 0.00% | 0.03% | 0.01% | 1.65% |
| CK35 | 86.12% | 0.17% | 7.98% | 0.10% | 0.00% | 4.81% | 0.00% | 0.00% | 0.00% | 0.81% |
| TA35 | 39.36% | 1.61% | 29.78% | 26.79% | 0.00% | 1.84% | 0.00% | 0.00% | 0.00% | 0.61% |
| TB35 | 78.17% | 4.40% | 3.47% | 6.74% | 0.00% | 6.37% | 0.00% | 0.01% | 0.01% | 0.83% |

CK, aerobic compost with no additive. TA, aerobic compost with microbiological inoculation. TB, aerobic compost with microbiological inoculum combined with 10% biochar.

**Table S5.** The relative abundances of top ten bacterial genera of aerobic compost processing with three treatments.

| Sample ID | *Lactobacillus* | *Bacillus* | *Caldicoprobacter* | *Clostridium_sensu_stricto* | *Corynebacterium* | *Fermentimonas* | *Halocella* | *Prevotella* | *Pseudogracilibacillus* | *Pseudomonas* | *Sinibacillus* | *Streptococcus* | *Tepidimicrobium* | *Terrisporobacter* | *uncultured_ Bacillaceae* |
| --- | --- | --- | --- | --- | --- | --- | --- | --- | --- | --- | --- | --- | --- | --- | --- |
| CK2 | 44.03% | 0.01% | 0.00% | 16.50% | 0.61% | 0.01% | 0.00% | 7.78% | 0.00% | 1.71% | 0.00% | 17.28% | 0.00% | 12.06% | 0.01% |
| TA2 | 48.71% | 0.00% | 0.00% | 16.41% | 0.18% | 0.01% | 0.00% | 13.41% | 0.00% | 1.58% | 0.00% | 7.77% | 0.01% | 11.90% | 0.01% |
| TB2 | 26.88% | 0.07% | 3.26% | 9.03% | 7.05% | 12.85% | 0.30% | 6.89% | 0.00% | 0.51% | 0.00% | 5.16% | 3.90% | 7.82% | 16.28% |
| CK8 | 35.65% | 0.01% | 0.01% | 31.69% | 1.09% | 0.01% | 0.00% | 0.13% | 0.00% | 2.75% | 0.00% | 0.67% | 0.15% | 27.83% | 0.02% |
| TA8 | 41.57% | 0.02% | 0.00% | 25.25% | 1.67% | 0.01% | 0.00% | 0.56% | 0.00% | 0.19% | 0.00% | 6.36% | 0.00% | 24.34% | 0.01% |
| TB8 | 40.90% | 0.01% | 0.00% | 10.57% | 35.51% | 0.13% | 0.00% | 0.48% | 0.00% | 0.08% | 0.00% | 2.53% | 0.00% | 9.77% | 0.01% |
| CK16 | 18.63% | 0.48% | 0.32% | 44.17% | 1.05% | 0.17% | 0.08% | 0.02% | 0.01% | 0.02% | 0.01% | 0.12% | 3.87% | 24.57% | 6.46% |
| TA16 | 1.45% | 0.40% | 7.33% | 6.42% | 3.12% | 7.08% | 1.19% | 0.01% | 0.01% | 0.03% | 0.00% | 0.23% | 12.07% | 3.66% | 57.00% |
| TB16 | 15.26% | 0.15% | 3.04% | 8.21% | 22.22% | 18.03% | 2.04% | 4.51% | 0.00% | 0.20% | 0.00% | 2.83% | 4.70% | 6.21% | 12.59% |
| CK26 | 2.58% | 32.32% | 1.88% | 21.19% | 0.31% | 0.51% | 0.57% | 0.01% | 1.04% | 0.02% | 16.74% | 0.03% | 6.82% | 11.98% | 3.99% |
| TA26 | 0.51% | 36.19% | 1.49% | 10.86% | 1.17% | 0.81% | 1.15% | 0.01% | 5.19% | 2.43% | 8.42% | 0.11% | 10.94% | 5.76% | 14.96% |
| TB26 | 1.08% | 30.90% | 2.66% | 6.02% | 30.79% | 0.68% | 4.07% | 0.00% | 0.43% | 0.02% | 6.95% | 0.23% | 6.74% | 5.37% | 4.07% |
| CK35 | 0.09% | 8.74% | 3.79% | 7.93% | 0.05% | 9.05% | 5.47% | 0.00% | 13.93% | 0.02% | 7.25% | 0.00% | 13.85% | 4.36% | 25.47% |
| TA35 | 0.07% | 5.75% | 2.88% | 2.70% | 0.08% | 34.69% | 2.15% | 0.00% | 7.11% | 27.26% | 1.46% | 0.02% | 4.49% | 1.53% | 9.80% |
| TB35 | 0.25% | 26.41% | 5.69% | 3.33% | 4.98% | 4.88% | 9.02% | 0.00% | 5.18% | 0.36% | 7.74% | 0.10% | 13.85% | 3.04% | 15.17% |

CK, aerobic compost with no additive. TA, aerobic compost with microbiological inoculation. TB, aerobic compost with microbiological inoculum combined with 10% biochar.

**Table S6.** Relationships between bacterial community compositions at genus level and physicochemical variables revealed by Mantel test.

| Factors | Mantel test | |
| --- | --- | --- |
|  | r | *P* |
| Temperature | 0.129 | **0.003** |
| pH | 0.197 | **0.003** |
| water | 0.412 | **0.001** |
| EC | 0.233 | **0.001** |
| TOC | 0.118 | **0.01** |
| TN | 0.422 | **0.001** |
| NO_3_^-^-N | 0.235 | **0.001** |
| NH_4_^+^-N | 0.162 | **0.001** |
| TK | 0.223 | **0.001** |
| TP | 0.088 | 0.032 |

Values in bold indicate significant correlations (*P* < 0.01). EC, electrical conductivity ;TN, total nitrogen; TOC, total organic carbon; NO_3_^-^-N, and NH_4_^+^-N, Nitrate Nitrogen and Ammonium Nitrogen; TP, Total phosphorus; TK, total potassium.

**Figure S1.** The schematic diagram of compost reactor.

**Figure S2.** Changes in germination index (GI) during 35 days of aerobic composting. CK, aerobic compost with no additive. TA, aerobic compost with microbiological inoculation. TB, aerobic compost with microbiological inoculum combined with 10% biochar.

**Figure S3.** Rarefaction curves based on 16S rRNA sequences among the different samples.

**Figure S4.** Correlation between bacterial alpha diversity and physicochemical variables for all samples. Asterisks indicate the statistical significance (****P* < 0.001; ***P* < 0.01; and **P* < 0.05).

**Figure S5.** Pearson correlation coefficients between bacterial community structure and physicochemical factors at genus level.

**Figure S6**. Ranking variable importance that between physicochemical factors and bacterial communities at the genus level by random forest. IncMSE, increase in

mean square error, IncNodePurity, increase in impurity at nodes.
