# Supplementary figures and images for: Different Effects of Thermophilic Microbiological Inoculation With and Without Biochar on Physicochemical Characteristics and Bacterial Communities in Pig Manure Composting
Source: Front Microbiol. 2021 Nov 16;12:746718. doi: 10.3389/fmicb.2021.746718 (PMC8660119; doi:10.3389/fmicb.2021.746718)

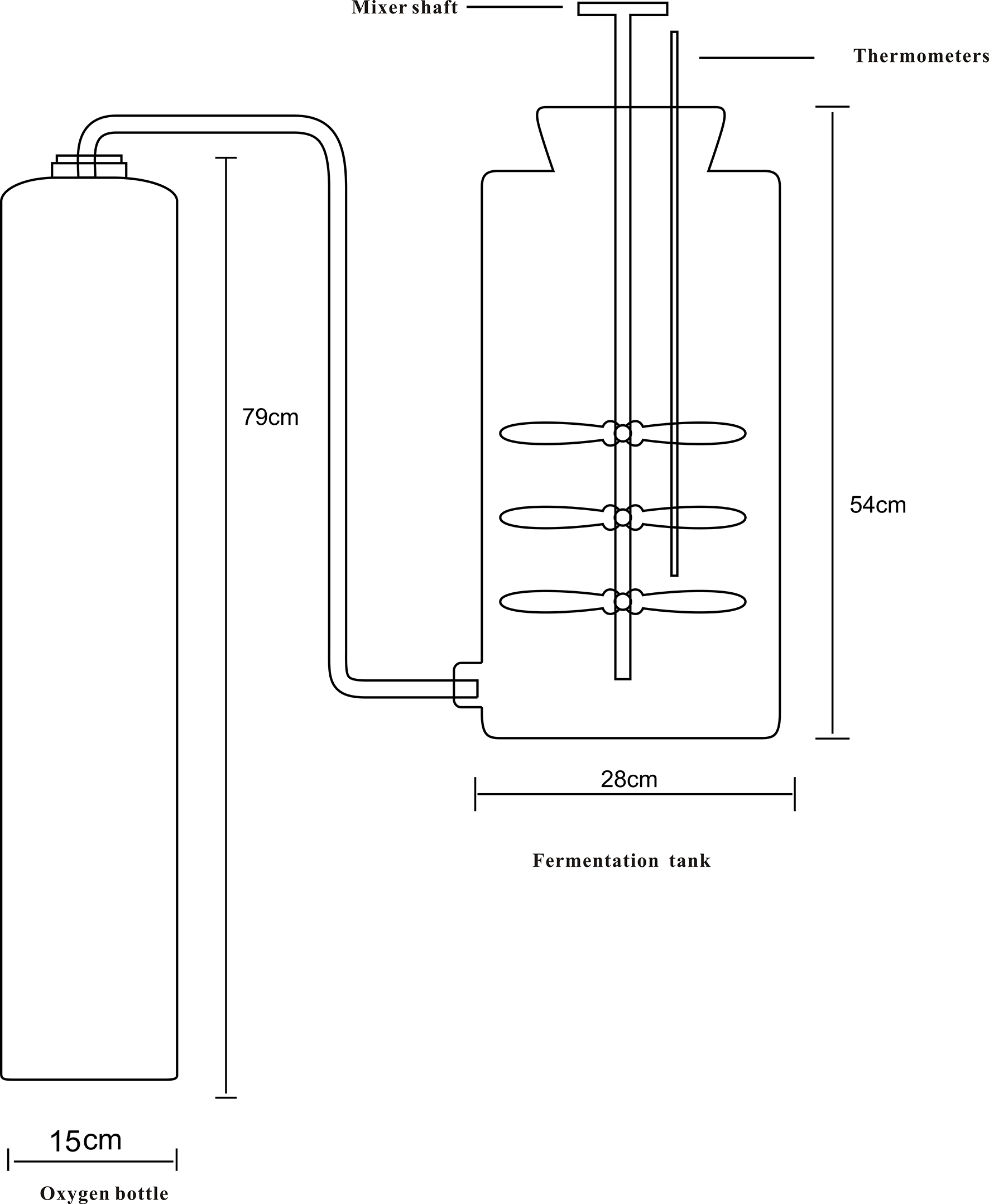

Supplement: Supplementary file 2 [file Image_1.TIF]

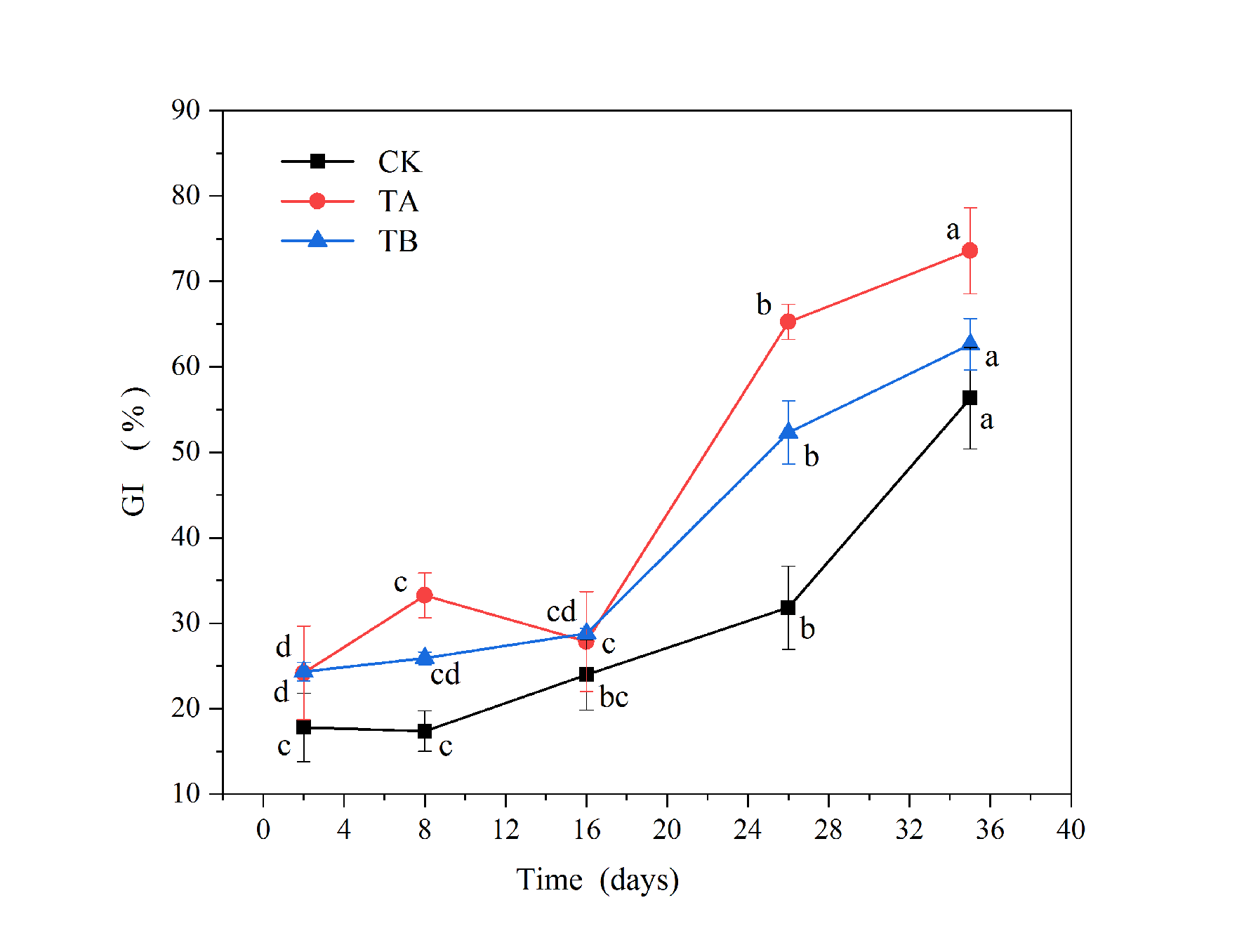

Supplement: Supplementary file 3 [file Image_2.TIF]

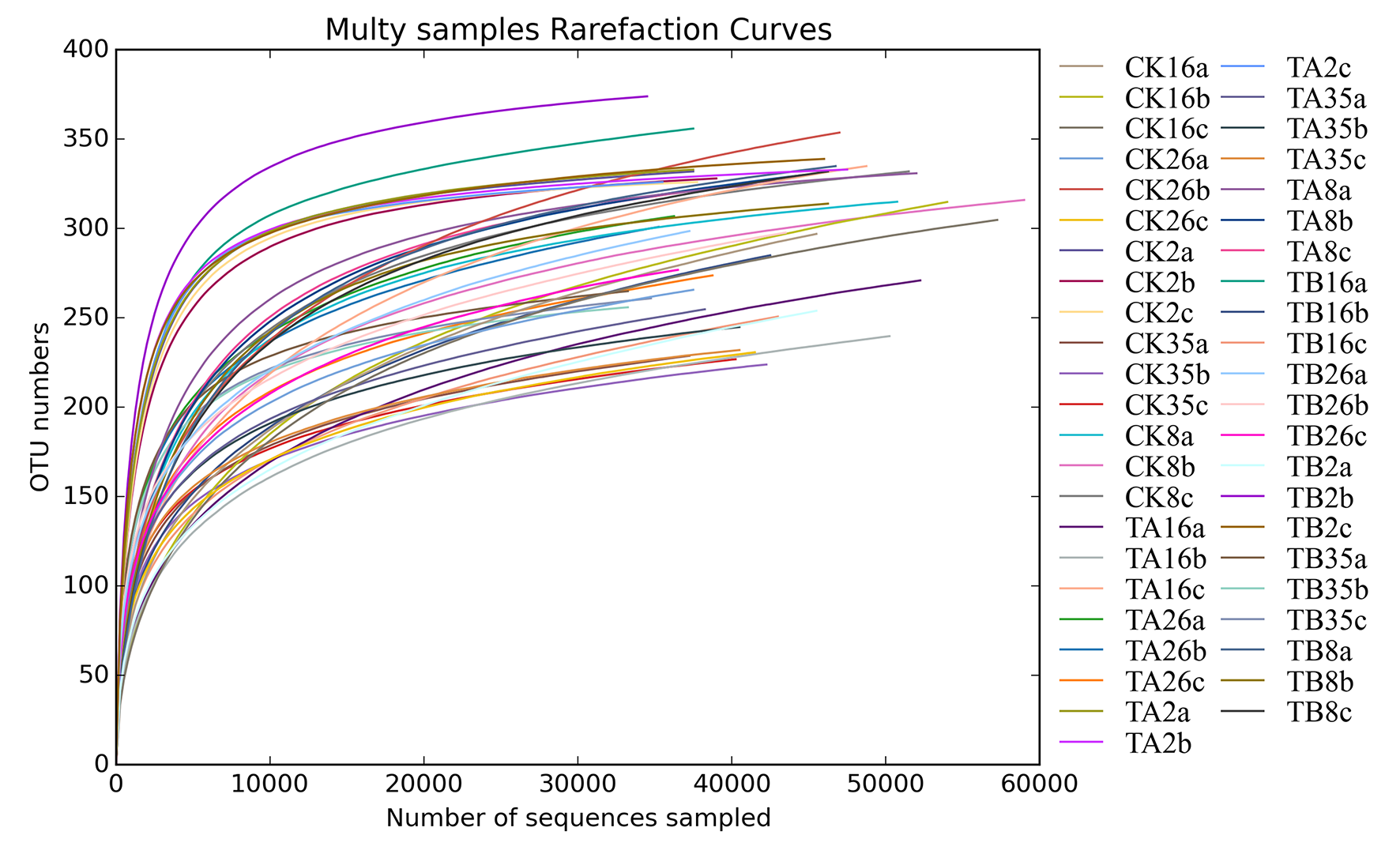

Supplement: Supplementary file 4 [file Image_3.TIF]

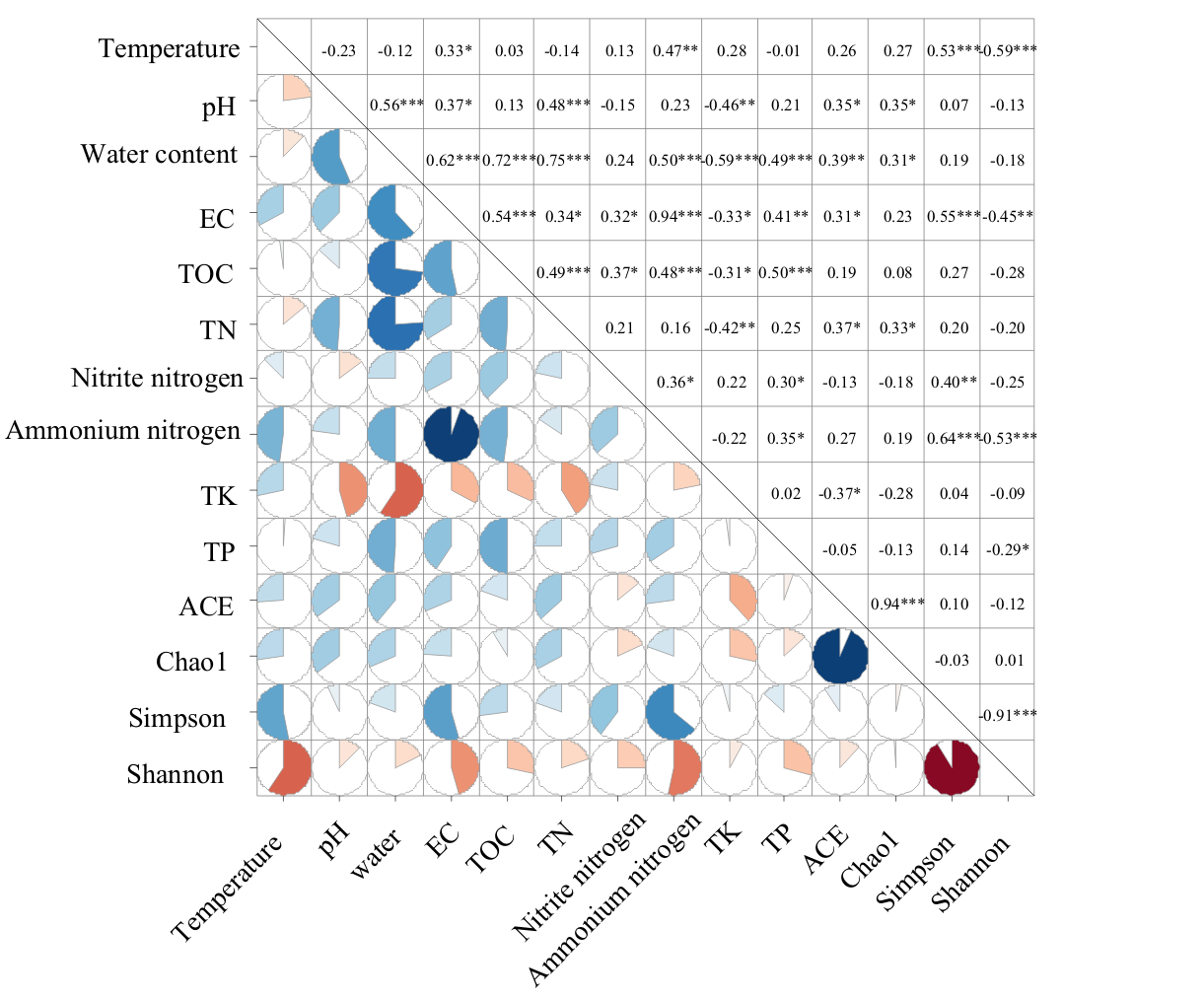

Supplement: Supplementary file 5 [file Image_4.TIF]

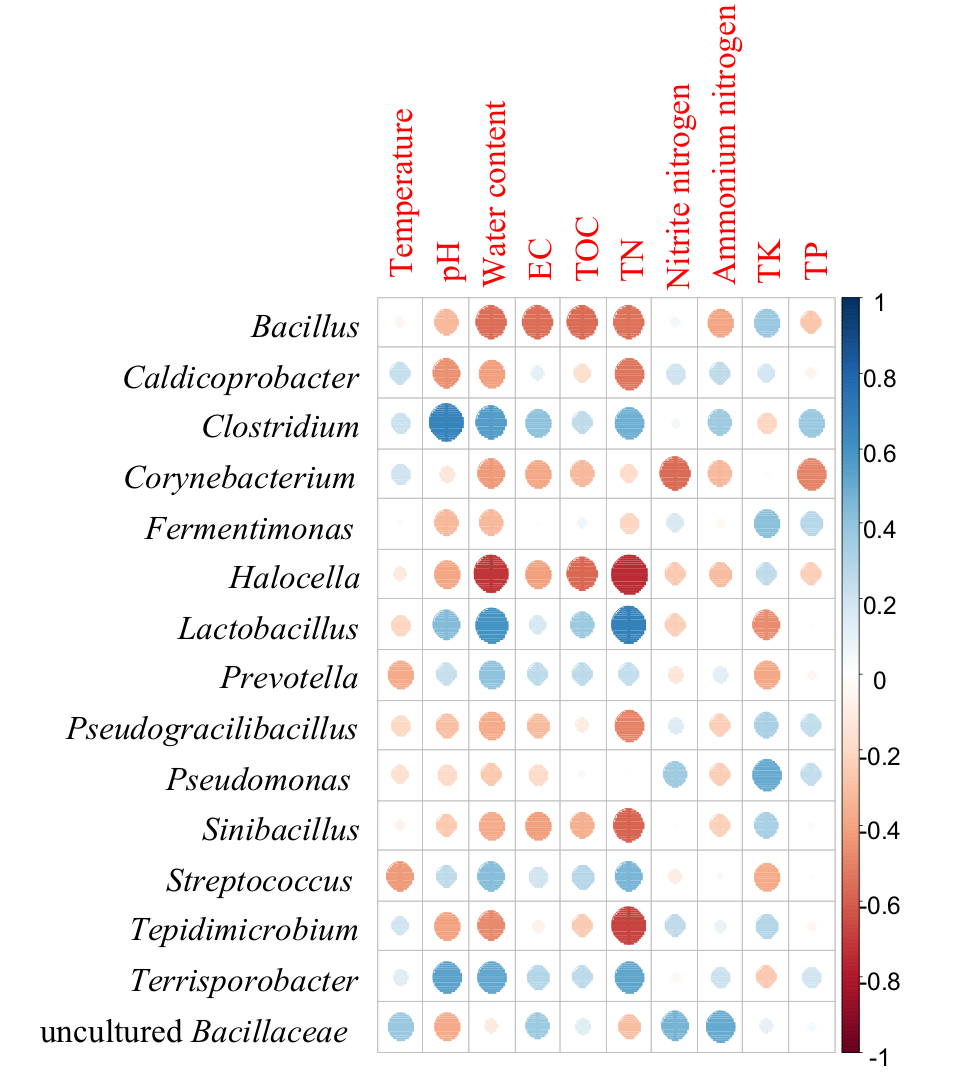

Supplement: Supplementary file 6 [file Image_5.TIF]

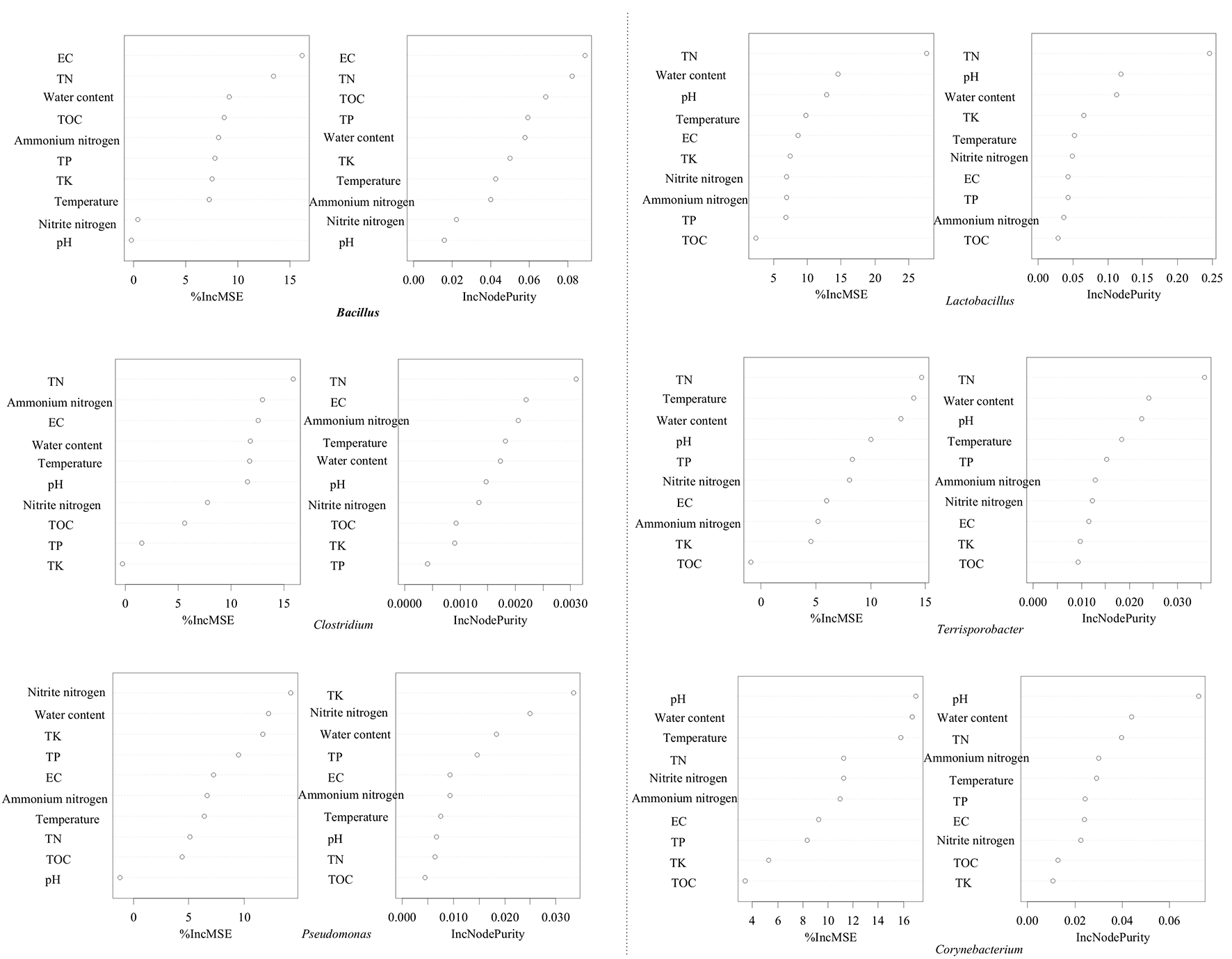

Supplement: Supplementary file 7 [file Image_6.TIF]
